# Immune-related disorders in families of children with inflammatory bowel disease - A prospective cohort study

**DOI:** 10.1186/1824-7288-37-49

**Published:** 2011-10-04

**Authors:** Alyzée M Sibtain, Donald Spady, Wael El-Matary

**Affiliations:** 1 Department of Pediatrics, Stollery Children's Hospital, Faculty of Medicine, University of Alberta, Edmonton, Canada; 2 Section of Pediatric Gastroenterology, Department of Pediatrics, University of Manitoba, Winnipeg, Canada

## Abstract

**Background:**

The aim of this paper was to examine the prevalence of immune-related disorders in families of children with inflammatory bowel disease (IBD) compared to those without IBD.

**Methods:**

Children ≤18 years of age presenting to the IBD clinic between September 2007 and August 2009 with an established diagnosis of IBD were recruited. Age and sex matched controls without IBD were recruited. The study was a single-centre prospective cohort study. Outcome measures were prevalence of immune-based/inflammatory diseases in families of both patients and controls.

**Results:**

One hundred and eight children in each group were recruited. Asthma was the most frequently reported disease in families of the IBD patients (52.8%) and controls (46.3%). The prevalence of IBD in families of IBD patients was significantly higher than in those without IBD (OR 2.03, 95% CI 1.04-3.95).

**Conclusions:**

The prevalence of immune-based disorders, as a group, in families of children with IBD was not significantly higher when compared to children without IBD.

## Background

Inflammatory bowel diseases (IBD), Crohn's disease (CD) and ulcerative colitis (UC) are considered to be gastrointestinal luminal diseases of immune-dysregulation [1,2]. It is postulated that, IBD results from dysfunctional luminal epithelial barrier, and facilitated by an inappropriate innate and acquired immune response to commensal microorganisms that occur in individuals with appropriate genetic predisposition and susceptibility [3,4]. A genome study by Becker *et al*. demonstrated the presence of non-random overlapping of susceptibility loci among autoimmune diseases such as multiple sclerosis (MS), CD, psoriasis, asthma and type I diabetes (IDDM) [5]. Furthermore, there is considerable evidence that other immune-related disorders may be associated with bronchial asthma and rheumatoid arthritis [6-9].

The aim of this single-centre, hospital-based prevalence study was to examine the prevalence of family history of immune-based diseases among pediatric patients with IBD compared to those without IBD. We hypothesized that there is an increased prevalence of inflammatory or immune-based disease among children diagnosed with IBD compared to children without IBD.

## Methods

In a single centre cohort prospective study, children ≤18 years of age presenting to the IBD clinic or emergency room, between September 2007 and August 2009, with an established diagnosis of IBD based on clinical, radiological and endoscopic evidence [10] were recruited (n = 108). During the same time, children ≤18 years presenting to the general gastrointestinal clinics with functional problems, gastroesophageal reflux or feeding problems, were recruited as controls (n = 108). IBD was excluded in the control group based on clinical, radiological, and endoscopy if indicated. Patients and controls were age and sex-matched. Family history of some immune-based and inflammatory disorders, including IBD, celiac, autoimmune thyroid disease (including Grave's and Hashimoto's), rheumatoid arthritis, bronchial asthma, autoimmune liver disease, multiple sclerosis (MS), and psoriasis or vitiligo from both patients and controls was collected during clinic visits.

A standardized questionnaire was used for each immune-based disease. Responses were provided from adults accompanying patients, most often patient's parents and recorded by the investigators during the interview. First-degree relatives were defined as parents and siblings of patients, and second-degree relatives were defined as uncles, aunts, and grandparents. For uncles and aunts it was clarified that the affected relative was a genetic relative, rather than related by marriage. All other relatives such as cousins and great grandparents were classified as distant relatives.

Measures were taken to reduce recall/interviews bias, included verification of the history taken during clinic visits against hospital records and taking family history every time patients or controls presented to IBD/GI clinics.

Informed consents were obtained from all participants after circulating study information sheets.

### Ethics

The study protocol was approved by the Health Research Ethics Board of the University of Alberta, Edmonton, Canada.

### Statistical analysis

Data were analyzed using Stata10 ^(TM) ^(Data Analysis and Statistical Software, Stata Corp, Austin, TX, USA). Summary statistics were obtained for all variables. Cross-tabulation analysis was made for all variables against disease (case/control status) to explore any possible relationships. An initial conditional logistic regression model was performed, with disease status (case or control) as the dependent variable and potential immune disorders as independent variables. Subsequently, only those variables that were significant at p < 0.05 level in the initial regression model were included in the final model.

## Results

Asthma was the most frequently reported disease in both IBD patients (52.8%) and the control group (46.3%), as well as when CD (52.5%) and UC (53.1%) patients were examined separately [Table 1]. The second most frequently reported immune-mediated disease in IBD group was IBD (33.3%), followed by autoimmune thyroid disease and rheumatoid arthritis (31%). When examined separately (CD and UC), the second most frequently reported immune-mediated disorder in patients with CD after bronchial asthma was autoimmune thyroid disease (30.5%), followed by rheumatoid arthritis (28.8%) and IBD (27.1%). In UC patients, the second most frequently reported immune-mediated disorder was IBD (40.8%), followed by rheumatoid arthritis (34.7%) and thyroid disease (32.7%). In the control group, the second most commonly reported immune-mediated disease was rheumatoid arthritis (33.3%), followed by IBD and thyroid disease (24.1%).

**Table 1:** The Prevalence of immune-related disease in families of patients with IBD vs. controls

	Diagnosis							
**Immune-Mediated Disorder**	**IBD (n = 108)**		**CD (n = 59)**		**UC (n = 49)**		**Control (n = 108)**	

	**N**	**%**	**N**	**%**	**N**	**%**	**N**	**%**

**Total Immune-Mediated**	89	82.4	47	79.7	42	85.7	92	85.2
**IBD**	36	33.3	16	27.1	20	40.8	23	21.3
**Celiac**	9	8.33	5	8.47	4	8.16	8	7.14
**IDDM**	30	27.8	16	27.1	14	28.6	26	24.1
**Thyroid**	34	31.5	18	30.5	16	32.7	45	24.1
**Parathyroid**	2	1.85	1	1.69	1	2.04	1	0.93
**Skin**	19	17.6	12	20.3	7	14.3	23	21.3
**Joint**	34	31.5	17	28.8	17	34.7	36	33.3
**Asthma**	57	52.8	31	52.5	26	53.1	50	46.3
**MS**	8	7.41	3	5.08	5	10.2	9	8.33
**Liver**	2	1.85	1	1.69	1	2.04	4	3.7

Abbreviations:

IBD inflammatory bowel disease, CD Crohn's disease, UC ulcerative colitis, MS multiple sclerosis, IDDM insulin-dependent diabetes mellitus

There was no statistically significant difference observed in the prevalence of immune-mediated diseases as a group in the family history of patients when compared to children without IBD [Table 2]. However, when the prevalence of IBD, celiac, thyroid disease, parathyroid disease, rheumatoid arthritis, bronchial asthma, autoimmune liver disease, MS, and psoriasis or vitiligo was examined individually, there was a statistically significant difference between IBD patients and controls, specifically in regards to IBD. The prevalence of IBD in the self-reported family history of IBD patients was higher than the prevalence of IBD in families of children without IBD (OR 2.03, 95% CI 1.04-3.95) and the difference becomes higher in families with UC patients (OR 5.3, 95% CI 1.5-18.67)

**Table 2:** Calculated statistical difference in the family history of immune-related disease in children with IBD

	IBD				CD				UC			
**Immune-Mediated Disease**	**Odds Ratio**	**95% CI**		**p-value**	**Odds Ratio**	**95% CI**		**p-value**	**Odds Ratio**	**95% CI**		**p-value**

**Total Immune-Mediated Disease**	0.82	0.40	1.67	0.71	0.90	0.37	2.20	1	0.68	0.21	2.21	0.76
**IBD**	2.03	1.04	3.95	*<0.05*	0.87	0.31	2.42	0.79	5.30	1.50	18.67	*<0.05*
**Celiac**	0.91	0.28	2.94	0.87	3.27	0.28	38.42	0.35	1.49	0.20	11.21	0.7
**IDDM**	1.37	0.69	2.72	0.37	1.63	0.60	4.47	0.34	1.95	0.58	6.60	0.28
**Thyroid**	0.57	0.29	1.07	0.08	0.71	0.30	1.70	0.44	0.29	0.08	1.023	0.05
**Parathyroid**	1.56	0.10	24.36	0.75	0.15	0.002	9.61	0.38	0	0	3.86	0.99
**Skin**	0.76	0.35	1.61	0.48	0.81	0.31	2.26	0.71	0.53	0.13	2.10	0.37
**Joint**	0.88	0.46	1.68	0.69	0.79	0.31	2.02	0.62	1.38	0.42	4.54	0.59
**Asthma**	1.32	0.75	2.30	0.34	1.84	0.87	3.89	0.11	1.08	0.34	3.37	0.9
**MS**	0.887	0.28	2.71	0.81	1.47	0.23	9.41	0.61	0.60	0.11	3.36	0.56
**Liver**	0.70	0.09	5.35	0.73	2.64	0.08	86.32	0.59	0.33	0.01	7.98	0.49

Abbreviations:

IBD inflammatory bowel disease, CD Crohn's disease, UC ulcerative colitis, MS multiple sclerosis, IDDM insulin-dependent diabetes mellitus

No statistically significant difference was observed in the prevalence of any of the other individually-examined immune-related diseases in families of children with IBD when compared to children without IBD.

The IBD patients were then categorized based on their diagnosis of CD or UC. There was no statistically significant difference in the prevalence of immune-mediated diseases as a group in families of children with UC or CD when compared to children without IBD.

## Discussion

Over 30% of older school children in Saskatchewan had wheezing in 12 months prior to a survey that was published in 1999 [11]. This prevalence of asthma in Canada is rising [12]. Asthma was the most frequently reported disease in families of children with IBD, similar to those without IBD. Interestingly, some reports indicated a higher prevalence of irritable bowel syndrome (a functional disorder) among young asthmatics [13].

Rheumatoid arthritis was the second most common disorder followed by IBD. This finding is consistent with results from previous studies examining co-morbid conditions among IBD patient [7,14,15]. In a cross-sectional study from Northern California, Weng *et al *identified asthma, rheumatoid arthritis and MS to be the most common co-morbidities among IBD patients, and suggested genetic linkage of immune-mediated diseases [16]. Thus, it is not unreasonable to suggest that these common co-morbidities as well as IBD may also be more common in families of pediatric patients with IBD.

The IBD occurrence in family of control group 24.1%. The prevalence of IBD worldwide is approximately 396/100,000 in a standard population [15]. This discrepancy between the prevalence in the control group and the standardized population may be due to the smaller sample size. Another explanation is the fact that the control group was recruited from patients with functional gastrointestinal problems rather than from general population.

The prevalence of self-reported immune-based disorders, as a group, in families of children with IBD was not significantly higher when compared to children without IBD. The relationship between autoimmune diseases and IBD has been examined, and several studies have shown an association between IBD and a positive family history specifically of MS, rheumatoid arthritis, and ankylosing spondylitis [16-18].

There was, on the other hand, a significantly higher prevalence of IBD in families of children with IBD, especially in children with UC compared to those without IBD. This finding is consistent with previous studies demonstrating a familial aspect of IBD [9,15,19]. A cohort study from six pediatric centres in the United States by Heyman *et al *reported a positive family history of IBD to be more common in UC patients [20]. However, there was not a significantly higher prevalence of IBD in families of children with CD. This is surprising, as CD is known to have a significant familial component as shown by genetic and population studies [8,19]. Nonetheless, a recent study by Hemminki *et al*. using The Multi-generation Register in Sweden demonstrated that, though present, concordant familial risks for both UC and CD were lower than previously cited [17].

The potential limitation of recall bias needs to be considered. Though patients were asked specifically about bronchial asthma, there are other types of wheezy chest syndromes that can be confusing. This may explain why asthma was not only the most commonly reported immune-mediated disease in both the patient and control group family histories, but also why there was no a significant difference in its prevalence between the two groups.

Measures taken to reduce recall/interviews bias, including verification of history taken against hospital records and taking family history very time patients or controls were presented to IBD/GI clinics.

Another limitation is that the control group was a group of children with functional gastrointestinal disorders like irritable bowel syndrome. It is better to recruit a control group with no gastrointestinal symptoms but there is no evidence to suggest that inflammatory bowel diseases are more prevalent in relatives of patients with functional gastrointestinal problems.

The presence of a significantly increased prevalence of positive family history of IBD in patients with IBD lends further support to the exploration of family history when determining the direction of investigations and diagnosis in children presenting with symptoms of IBD, as well as future screening and follow up is warranted.

## Conclusions

The present study supports the available evidence documenting a significantly increased prevalence of positive family history of IBD, among pediatric IBD patients compared to children without IBD. However, the prevalence of self-reported immune-based disorders, as a group, in families of children with IBD was not significantly higher when compared to children without IBD.

## Abbreviations

IBD:inflammatory bowel disease; CD: Crohn's disease; UC: ulcerative colitis; MS: multiple sclerosis; IDDM: insulin-dependent diabetes mellitus.

## Competing interests

The author declares that they have no competing interests.

## Authors' contributions

AS interviewed patients and their families and wrote the manuscript. DS performed the statistical analysis. WEM conceived of the study, participated in its design and coordination, and helped to draft the manuscript. All authors read and approved the final manuscript
